# Avoidable mortality does not allow valid conclusions on population health and health system quality in Western Europe—authors’ reply

**DOI:** 10.1016/j.lanepe.2025.101213

**Published:** 2025-01-27

**Authors:** Rok Hrzic, Tobias Vogt, Pauline Pearcy, Ilias Kyriopoulos, Elias Mossialos

**Affiliations:** a Department of International Health, Care and Public Health Research Institute (CAPHRI), Maastricht University, 6200 MD, Maastricht, the Netherlands; b Population Research Centre, Faculty of Spatial Sciences, University of Groningen, 9700 AV, Groningen, the Netherlands; c Prasanna School of Public Health, Manipal Academy of Higher Education, Karnataka, 576104, India; d Department of Health Policy, London School of Economics and Political Science, London, UK

In their letter,^1^ Stolpe and Kowall raise concerns about the validity of using cause-specific mortality rates for health system performance assessment (HSPA) in our studies.^2^^,^^3^ They claim that differences in cause-specific mortality, particularly for cardiovascular disease, do not accurately reflect morbidity differences due to (1) subjective and context-dependent death certification misclassifying avoidable deaths and (2) varied causal chains leading to death potentially involving multiple diseases.

We find the claim that avoidable mortality is an inaccurate indicator unwarranted. Avoidable mortality reflects deaths avoidable through public health interventions or medical treatment. Evidence shows the life expectancy gap in the European Union stems from deaths caused mainly by harmful health behaviours. Our study indicates that addressing avoidable risk factors can reduce mortality disparities within and across EU countries.^2^ Hence, the letter's claims seem based primarily on a critique of amenable mortality related to cardiovascular disease. While this concern is important, it is essential to note that amenable mortality also includes many causes of death with clear causal pathways.

Analysing differences in cardiovascular mortality, the largest category of avoidable deaths in Europe, is critical for understanding mortality differences and trends in the region. Large-scale misclassification of cardiovascular deaths could jeopardise this research. While challenges with death certification exist in high-income areas, the evidence does not suggest widespread misclassification.^4^ Our sensitivity analyses found a limited impact of ill-defined causes of death on avoidable cardiovascular mortality rates. Nonetheless, we agree that further nationally representative and internationally comparable studies are required to adequately assess death certification bias and its impact on comparative mortality research.

Avoidable mortality is a useful yet imperfect indicator of health system performance, as acknowledged in both studies. No single measure can perfectly capture the complexities of health system effectiveness; therefore, performance must be assessed using a range of complementary indicators.^5^ Furthermore, establishing an unequivocal causal chain between access to high-quality preventive and curative services and deaths remains challenging. Including data on cause-specific morbidity to examine avoidable morbidity and avoidable case fatality would be a step forward. Including secondary causes of death could also help. Unfortunately, the health data ecosystem lacks the required data across many European countries. We hope that initiatives such as the European Health Data Space and other initiatives to promote the use of big data in Europe will address these gaps and improve data availability in the coming years.^6^

However, the list of causes considered avoidable may also need to be reconsidered. Previous studies have suggested devising more context-sensitive lists and updating them frequently to keep up with the pace of medical innovation.^7^ To improve avoidable mortality as an HSPA indicator, public health researchers can work closely with clinical practitioners and health policymakers to identify causes of death and ages that best reflect the provision of high-quality preventive and curative care.

Despite its limitations, analysing trends in avoidable (cardiovascular) mortality, compared to all-cause mortality, has provided useful evidence and important policy insights into health systems performance trajectories and remains an indispensable tool for comparative health systems research.

## Contributors

All authors contributed equally to the manuscript.

## Declaration of interests

We declare no competing interests.

## References

[1] Stolpe S., Kowall B. (2025). Avoidable mortality does not allow valid conclusions on population health and health system quality in Western Europe. Lancet Reg Health Eur.

[2] Hrzic R., Vogt T. (2024). The contribution of avoidable mortality to life expectancy differences and lifespan disparities in the European Union: a population-based study. Lancet Reg Health Eur.

[3] Cherla A., Kyriopoulos I., Pearcy P. (2024). Trends in avoidable mortality from cardiovascular diseases in the European Union, 1995–2020: a retrospective secondary data analysis. Lancet Reg Health Eur.

[4] Mikkelsen L., Iburg K.M., Adair T. (2020). Assessing the quality of cause of death data in six high-income countries: Australia, Canada, Denmark, Germany, Japan and Switzerland. Int J Public Health.

[5] Smith P.C., Mossialos E., Papanicolas I., Leatherman S. (2010). Performance Measurement for Health System Improvement: Experiences, Challenges and Prospects.

[6] Salas-Vega S., Haimann A., Mossialos E. (2015). Big data and health care: challenges and opportunities for coordinated policy development in the EU. Health Syst Reform.

[7] Pérez G., Rodríguez-Sanz M., Cirera E., Pérez K., Puigpinós R., Borrell C. (2014). Commentary: approaches, strengths, and limitations of avoidable mortality. J Public Health Policy.

